# Outcomes of pregnancies with trisomy 16 mosaicism detected by NIPT: a series of case reports

**DOI:** 10.1186/s13039-021-00559-w

**Published:** 2021-09-20

**Authors:** Haishan Peng, Jiexia Yang, Dongmei Wang, Fangfang Guo, Yaping Hou, Aihua Yin

**Affiliations:** Prenatal Diagnosis Centre, Maternal and Children Metabolic-Genetic Key Laboratory, Guangdong Women and Children Hospital, 521-523 Xingnan Road, Guangzhou, 511442 Guangdong China

**Keywords:** Low birth weight, CMA, Mosaic trisomy 16 (MT16), Noninvasive prenatal testing (NIPT), Prenatal diagnosis

## Abstract

**Background:**

Trisomy 16 (T16) is thought to be the most frequent chromosome abnormality at conception, which is often associated with a high risk of abnormal outcomes.

**Methods:**

A retrospective analysis of 14 cases with high risk of T16 by noninvasive prenatal testing (NIPT) was conducted. All cases in the analysis involved prenatal diagnosis, karyotyping and chromosomal microarray analysis.

**Case reports:**

NIPT detected 12 cases of T16 and 2 cases of T16 mosaicism. Prenatal diagnosis confirmed 5 true positive cases and 9 false positive cases. Among the 5 true positive cases, 3 cases had ultrasound abnormalities. All of the 9 false positive cases continued their pregnancies. The newborns who were from these 9 false positive cases except 1 case (case 7) had low birth weights (< 2.5 kg) and there were also 2 premature deliveries.

**Conclusion:**

NIPT serves as a fast and early prenatal screening method, giving clues to chromosome abnormalities and providing guidance for managing pregnancy. Confined placental mosaicism in 16 pregnancies may be at higher risk for preterm delivery.

## Introduction

In 2011, noninvasive prenatal testing (NIPT) was introduced to clinical practice, and the application of this technology has continuously evolved. Recently, researchers have started to focus on sharing their experience with expanded NIPT and discussing the outcomes of rare autosomal trisomies (RAT, defined as any autosomal trisomy other than T21, T18, and T13). Trisomy 16 (T16) is of particular importance as it is thought to be the most frequent chromosome abnormality at conception [1], with an incidence of ~ 1.5% [2]. Complete T16 is incompatible with life, while viable mosaic trisomy 16 (MT16) has been reported extensively in the literature [3, 4]. Almost all pregnancies which involve MT16 originate from a trisomy 16 zygote because of maternal meiosis I nondisjunction [5]. As with many trisomic conceptuses, trisomy 16 mosaicism can undergo rescue, with the risk of residual mosaicism and uniparental disomy (UPD) for chromosome 16 in the surviving fetus. UPD is the inheritance of both homologs of a chromosome from only one parent with no representative copy from the other parent, and UPD 16 is the most common [6].

In addition to UPD, other factors that may contribute to the pathogenesis of trisomy 16 mosaicism are (1) the degree of trisomy in various tissues of the placenta and fetal membranes; (2) the degree and distribution of trisomy in tissues of the fetus; and (3) the sex of the fetus [7].

MT16 is most likely associated with adverse perinatal outcomes, such as a high risk of abnormal outcomes, intrauterine growth retardation (IUGR), fetal death in utero, preeclampsia, preterm delivery, neonatal death, developmental delay, congenital heart defects, and other anomalies [4]. Thus, a detailed account of the detection of trisomy 16 is essential for numerous prenatal testing modalities. In this paper, we report a series of new cases of T16, perform a careful prenatal cytogenetic diagnosis for patients and provide more knowledge for reference about T16 and prenatal diagnoses for clinicians.

## Materials and methods

### Detection of NIPT

Whole blood samples of 5 to 10 ml from pregnant women were collected in EDTA tubes and processed according to the following procedures after collection within 8 h. Afterwards, cfDNA extraction, library construction, quality control, and pooling were performed by means of JingXin Fetal Chromosome Aneuploidy (T21, T18, and T13) Testing Kits (CFDA registration permit No. 0153400300). Then, the JingXin BioelectronSeq 4000 System (CFDA registration permit NO. 20153400309), a semiconductor sequencer, was used for DNA sequencing. Sequencing reads were filtered and aligned to the human reference genome (hg19) [8]. Fetal and maternal chromosome copy number variations (CNVs) were classified with our modified Stouffer’s z-score method as described previously [9]. Additionally, an absolute Z-score greater than 3 was marked with chromosome aneuploidies or microdeletions/microduplications.

### Prenatal diagnosis

NIPT high-risk cases were advised to undergo invasive prenatal diagnosis, including chromosome karyotype analysis and chromosomal microarray analysis (CMA). Metaphase chromosome G-banding karyotyping was performed at a level of 320 to 400 bands. CMA was performed for amniotic fluid or cord blood. Fetal genomic DNA was amplified, labeled, and hybridized by using the CytoScan 750 K array platform (Affymetrix, USA) according to the manufacturer’s protocol. Data were visualized by scanning with CytoScan™ and analyzed with Chromosome Analysis Suite software (Affymetrix, USA) based on the GRCH37 (hg19) assembly.

## Case reports

### NIPT and prenatal diagnosis results

A total of fourteen chromosome 16 abnormalities cases predicted by NIPT were included in this study. The age of the pregnant women was 23–43 years, and the gestational week was 13–25 weeks. The Z score of Chr. 16 was from 7.791–31.503, and the NIPT results predicted 12 cases of T16 and 2 cases of T16 mosaicism. The physician suggested that all high-risk pregnant women undergo prenatal diagnosis, including karyotyping and CMA. Prenatal diagnosis revealed 5 true positive cases (cases 1–5) and 9 false positive cases (cases 6–14). It is worth mentioning that cases 1–5 had T16 predicted by NIPT, but prenatal diagnosis confirmed that they had T16 mosaicism. The CMA result of case 5 showed two losses of heterozygosity (LOH) with deletion fragment sizes of 20.4 Mb and 6.1 Mb (Table 1).

**Table 1:** Prenatal diagnosis results of fourteen T16 cases predicted by NIPT

Case	Age	Gestational week at NIPT	NIPT Z score	NIPT result	Prenatal diagnosis	
					karyotype	CMA
1	43	13 +	11.345	T16	mos47,XN, + mar[8]/46,XN[14]	arr[hg19]4q34.3(178130290–179860825)*3 16p11.2q11.2(30995273–46786489)*3
2	35	18	17.824	T16	mos47,XN, + 16(4)/46,XN(17)	arr(16) × 2–3
3	32	18 +	31.503	T16	mos47,XN, + 16(1)/46,XN(19)	arr(16) × 2–3
4	28	25	8.629	T16	mos47,XN, + 16(1)/46,XN(80)	arr(16) × 2–2.3
5	36	17 +	9.317	T16	/	LOH in 16p13.3-p12.3 and 16q23.3-q24.3
6	23	16	17.542	T16	46,XN	arr(1–22) × 2
7	29	21 +	7.791	T16	46,XN	arr(1–22) × 2
8	34	13	13.304	T16	/	arr(1–22) × 2
9	27	19 +	7.874	T16	46,XN	/
10	33	18 +	22.812	T16	46,XN	arr(1–22) × 2
11	31	13	22.295	T16	/	arr(1–22) × 2
12	39	17	9.33	T16 mosaicism	46,XN	/
13	30	15	15.14	T16	/	arr(1–22) × 2
14	26	18	7.98	T16 mosaicism	46,XN	/

### Pregnancy outcomes

**Fig. 1:**
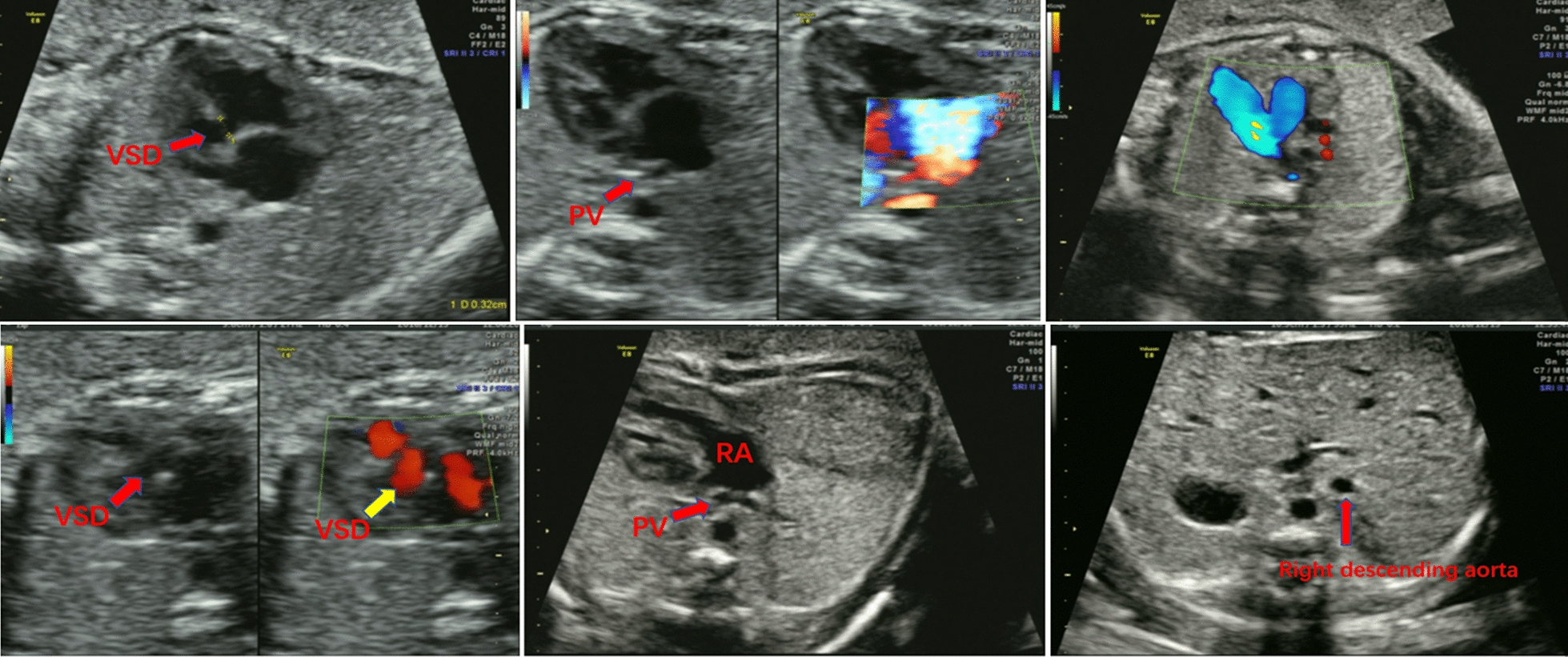
Echocardiography examination of case 2

Among the 5 true positive cases, case 1 insisted on continuing her pregnancy. Ultrasound examination suggested that case 1 had intrauterine growth restriction, a persistent right umbilical vein, and abnormal umbilical blood flow. In case 1, the baby was born with a low birth weight of 1.9 kg, and there were no other abnormalities by newborn screening. Both the karyotype and CMA showed T16 mosaicism in case 2. A three-stage ultrasound scan was carried out at 30 gestational weeks, and the results showed small limbs, a cardiac defect, and inconsistency with the gestational age. Further echocardiography examination showed total anomalous pulmonary venous drainage (TAPVD), a ventricular septal defect (VSD), and a left aortic arch with right descending aorta (Fig. 1). The parents were determined to continue the pregnancy. A male newborn was delivered by cesarean section at 37 gestational weeks. However, the infant died due to congenital heart disease 13 days after birth. In case 3, T16 mosaicism was confirmed. A prenatal three-stage ultrasound scan showed a butterfly vertebral anomaly in T3. At 30 gestational weeks, the pregnant woman underwent an MRI scan, which showed the same abnormality as the ultrasound result. The pregnancy was terminated. T16 mosaicism was also confirmed in case 4. The karyotype of the parental peripheral blood was normal. This pregnancy was also terminated. Case 5 was confirmed to have LOH at 16p13.3-p12.3 and 16q23.3-q24.3, and the fetus died in utero (Table 2).

**Table 2:** Pregnancy outcome of fourteen cases

Case	Ultrasound abnormality	Pregnancy outcome	Weight at birth	Newborn abnormalities	Other
1	IUGR, Persistent right umbilical vein, Abnormal cord blood flow	Continue pregnancy	1.9 kg	/	/
2	Abnormal heart	Died after birth	/	Congenital heart disease	/
3	Butterfly Cone	Induced labor	/	/	/
4	/	Induced labor	/	/	/
5	/	Intrauterine death, induced labor	/	/	UPD16
6	/	Continue pregnancy	2.1 kg	/	/
7	/	Continue pregnancy	3.1 kg	/	/
8	/	Continue pregnancy	1.55 kg	Anemia, cerebral edema	Preeclampsia
9	/	Continue pregnancy	1.75 kg	/	/
10	/	Continue pregnancy	1.75 kg	/	/
11	/	Continue pregnancy	2.2 kg	/	/
12	/	Continue pregnancy	2.1 kg	/	/
13	/	Continue pregnancy, premature delivery	1.7 kg	/	/
14	/	Continue pregnancy, premature delivery	2.2 kg	/	/

Among the 9 false-positive cases, all the pregnancies continued, and all babies, except in case 7, were born with low birth weights (< 2.5 kg). There were two premature deliveries, which suggested that trisomy 16 pregnancies may be at higher risk for preterm delivery. In addition, the baby in case 8 showed cerebral edema and anemia during newborn screening, and the mother had preeclampsia. The women in cases 13 and 14 delivered premature babies (Table 2).

## Discussion

Trisomy 16 is one of the most frequently encountered rare autosomal abnormalities in first-trimester abortion. Complete trisomy 16 is not compatible with life, so almost all cases of trisomy 16 are mosaic type. Fetuses with trisomy 16 mosaicism are viable and reported extensively in the context of prenatal diagnosis, but trisomy 16 mosaicism is associated with adverse pregnancy outcomes [7, 10]. For clinical counseling, it is essential to detect trisomy 16 mosaicism accurately.

The vast majority of chromosomal abnormalities can be detectable in the first trimester. Over the last few decades, with the development of technique, the classical combined first trimester test, screening for common chromosomal abnormalities at first trimester improved with more than 90% detection rates and 3–5% false positive rates, it is considered as “gold standard”. However, the introduction of noninvasive prenatal testing challenged this situation [11]. It is reported that NIPT had a detection rate about 99% and a false positive rate < 0.1% when screening for trisomy 21, 18, and 13 [12]. From 2015 to 2019, 44,423 pregnant women underwent NIPT tests in our prenatal diagnostic center, with a total false positive number of 66 and a total false negative number of 2. NIPT used in this study was performed by a semiconductor sequencing platform (SSP); the overall sensitivity and specificity of this platform for detecting trisomy 21, 18, and 13 combined were 99.61% and 99.91%, respectively [8].

Moreover, NIPT can offer information on other chromosomal abnormalities. Recent research proves that the ability of First trimester screening (FTS) to screen rare chromosomal abnormalities is poor [13]. Thus, as a fast, more accurate, and non-invasive method, NIPT would be preferred among patients. NIPT based on cell-free DNA is a new sequencing technique. It is available for use in pregnancies with a gestational time of 12 weeks or higher. In the present study, the earliest gestational NIPT to predict a high risk of T16 was performed at 13 weeks, which meant that NIPT could serve as a fast and early prenatal screening tool to provide information about chromosome abnormalities.

A previous study reported that the birth weights of live births from mosaic trisomy 16 pregnancies were below the gestational age-corrected mean birth weights in the general population [7, 14]. In our study, a true mosaic trisomy 16 pregnancy was continued, and the baby was born with a weight of 1.9 kg, which is low birth weight compared to average. Of the 9 false positive cases, all the pregnancies were continued, and all infants, except case 7, were born with low birth weights. This indicates that some level of below-average growth is a nearly universal phenomenon in trisomy 16 mosaicism and supports the hypothesis of undetected trisomy mosaicism as an etiological factor in both severe and mild idiopathic intrauterine growth restriction. In addition, there were 2 premature babies in this study, which suggests that trisomy 16 pregnancies may be at higher risk for preterm delivery. Further research is needed to determine whether there is truly a higher risk of preterm delivery in an unbiased population.

Cell-free DNA comes from the apoptotic cells of cytotrophoblasts, so the NIPT result shows a high risk of trisomy 16, indicating some trisomic cells in the placenta. It is recognized that the primary source of false positive results are confined placental mosaicism (CPM). CPM is a type of chromosomal mosaicism in which chromosome abnormalities are present in chorionic villi/placenta but not in the fetus itself. In our study, 9 cases were false positive, and we highly suspected that the CPM caused these. However, this study was a retrospective study, and all pregnancies had been completed. We could not obtain placental samples to verify our speculation, which was a limitation of our research. T16 was associated with poor outcomes[15]. Diane Van O and colleagues found that cases with abnormal NIPT T16 results most likely caused by placental aberrations manifest mostly as IUGR or small for gestational age (SGA) [16]. Another study by Yi H et al. showed that IUGR could be found in 20% of CPM cases for T16 detected by NIPT with placenta evidence [17]. In our series of cases, all false positive cases, except case 7, were low-birth-weight babies. We speculated that low birth weight was also related to CPM. However, more samples are needed for verification, which will also be one of our future research directions.

Case 5 had two LOHs: one in 16p13.3-p12.3 and another in 16q23.3-q24.3. The deletion sizes of the fragments were 20.4 Mb and 6.1 Mb. There was no evidence that the two LOHs in this area were pathogenic. Still, if there were recessive genetic disease-carrying genes in this region, the risk of recessive genetic disease would increase.

NIPT detected T16 or T16 mosaicism in this present study, with a true positive rate of 35.7% (5/14). In recent years, many studies have reported NIPT screened for other chromosome aneuploidy [18], but the PPVs for other chromosome aneuploidy were relatively low [19]. On one hand, these aneuploidies are less prevalent. On the other hand, many of them have high CPM. In CPM cases, the cytogenetic abnormality is confined to the placenta. Thus, CPM of T16 presents karyotype discordance between fetus and placenta. [20]. If there are placental samples to verify NIPT, it would be better. But in this retrospective study, we cannot obtain a placenta sample, which is a shortcoming of our ability to investigate this phenomenon.

Pregnant women choosing NIPT should be well informed of the accuracy, reliability, false-positive and false-negative rates. In addition, NIPT is a screening test, American College of Medical Genetics and Genomics (ACMG) is strongly suggested to confirm by invasive prenatal diagnosis for all positive findings [21]. Although IUGR or small for gestational age is a universal phenomenon in trisomy 16 mosaicism and CPM, the long-term neurodevelopment outcomes were reported favorable [22]. In this research, the outcome of 9 false positive cases is better than true trisomy 16 mosaicism cases, which means if confirmed tests were negative, the outcome might be promising. T16 CPM needs to be monitored closely for obstetric and neonatal complications. Results of MT16 should be delivered in a timely, objective, and nondirective manner for parents to make a decision.

In conclusion, trisomy 16 mosaicism is complex. NIPT serves as a fast and early prenatal screening method to give clues to chromosome abnormalities, guiding pregnancy management. Combined cytogenetic techniques and molecular methods can accurately detect trisomy 16 mosaicism. Confined placental mosaicism in T16 pregnancies may be at higher risk for preterm delivery.

## Data Availability

The datasets used and/or analyzed during the current study are available from the corresponding author on reasonable request.

## Abbreviations

ACMG: American College of Medical Genetics and Genomics
CMA: Chromosomal microarray analysis
CNVs: Copy number variations
CPM: Confined placental mosaicism
CV: Chorionic villu
CVS: Chorionic villus sampling
FTS: First trimester screening
IUGR: Intrauterine growth retardation
GA: Gestational age
LOH: Loss of heterozygosity
MT16: Mosaic trisomy 16
NIPT: Noninvasive prenatal testing
RAT: Rare autosomal trisomies
SCA: Small for gestational age
SSP: Semiconductor sequencing platform
T16: Trisomy 16
UPD: Uniparental disomy
TAPVD: Total anomalous pulmonary venous drainage
VSD: Ventricular septal defect

## References

[1] Wolstenholme J (1995). An audit of trisomy 16 in man. Prenat Diagn.

[2] Hassold TJ, Jacobs PA (1984). Trisomy in man. Annu Rev Genet.

[3] Kalousek DK (1994). Variable clinical expression of mosaic trisomy 16 in the newborn infant. Am J Med Genet.

[4] Chareonsirisuthigul T, Worawichawong S, Parinayok R, Promsonthi P, Rerkamnuaychoke B (2014). Intrauterine growth retardation fetus with trisomy 16 mosaicism. Case Rep Genet.

[5] Robinson WP, Barrett IJ, Bernard L, Telenius A, Bernasconi F, Wilson RD, Best RG, Howard-Peebles PN, Langlois S, Kalousek DK (1997). Meiotic origin of trisomy in confined placental mosaicism is correlated with presence of fetal uniparental disomy, high levels of trisomy in trophoblast, and increased risk of fetal intrauterine growth restriction. Am J Hum Genet.

[6] Nakka P, Pattillo Smith S, O'Donnell-Luria AH, McManus KF, Mountain JL, Ramachandran S, Sathirapongsasuti JF (2019). Characterization of prevalence and health consequences of uniparental disomy in four million individuals from the general population. Am J Hum Genet.

[7] Yong PJ, Barrett IJ, Kalousek DK, Robinson WP (2003). Clinical aspects, prenatal diagnosis, and pathogenesis of trisomy 16 mosaicism. J Med Genet.

[8] Hu H, Liu H, Peng C, Deng T, Fu X, Chung C, Zhang E, Lu C, Zhang K, Liang Z (2016). Clinical experience of non-invasive prenatal chromosomal aneuploidy testing in 190,277 patient samples. Curr Mol Med.

[9] Yin AH, Peng CF, Zhao X, Caughey BA, Yang JX, Liu J, Huang WW, Liu C, Luo DH, Liu HL (2015). Noninvasive detection of fetal subchromosomal abnormalities by semiconductor sequencing of maternal plasma DNA. Proc Natl Acad Sci USA.

[10] Pertile MD, Halks-Miller M, Flowers N, Barbacioru C, Kinnings SL, Vavrek D, Seltzer WK, Bianchi DW (2017). Rare autosomal trisomies, revealed by maternal plasma DNA sequencing, suggest increased risk of feto-placental disease. Sci Trans Med.

[11] Kagan KO, Sonek J, Wagner P, Hoopmann M (2017). Principles of first trimester screening in the age of non-invasive prenatal diagnosis: screening for chromosomal abnormalities. Arch Gynecol Obstet.

[12] Gil MM, Accurti V, Santacruz B, Plana MN, Nicolaides KH (2017). Analysis of cell-free DNA in maternal blood in screening for aneuploidies: updated meta-analysis. Ultrasound Obstet Gynecol.

[13] Kevin S, Mark DP, Leonard B (2014). First trimester detection of trisomy 16 using combined biochemical and ultrasound screening. Prenat Diagn.

[14] Usher R, McLean F (1969). Intrauterine growth of live-born Caucasian infants at sea level: standards obtained from measurements in 7 dimensions of infants born between 25 and 44 weeks of gestation. J Pediatr.

[15] Grati FR, Ferreira J, Benn P, Izzi C, Verdi F, Vercellotti E, Dalpiaz C, D'Ajello P, Filippi E, Volpe N (2020). Outcomes in pregnancies with a confined placental mosaicism and implications for prenatal screening using cell-free DNA. Genet Med.

[16] Van Opstal D, van Maarle MC, Lichtenbelt K, Weiss MM, Schuring-Blom H, Bhola SL, Hoffer MJV, Huijsdens-van Amsterdam K, Macville MV, Kooper AJA (2018). Origin and clinical relevance of chromosomal aberrations other than the common trisomies detected by genome-wide NIPS: results of the TRIDENT study. Genet Med.

[17] He Y, Liu YH, Xie RG, Liu SA (2019). Obstetrics DZLJUi, Gynecology: Rare autosomal trisomies on non-invasive prenatal testing: not as adverse as expected. Ultrasound Obstetrics Gynecol.

[18] Li H, Lei Y, Zhu H, Luo Y, Qian Y, Chen M, Sun Y, Yan K, Yang Y, Liu B (2018). The application of NIPT using combinatorial probe-anchor synthesis to identify sex chromosomal aneuploidies (SCAs) in a cohort of 570 pregnancies. Mol Cytogenet.

[19] Liu Y, Liu H, He Y, Xu W, Ma Q, He Y, Lei W, Chen G, He Z, Huang J (2020). Clinical performance of non-invasive prenatal served as a first-tier screening test for trisomy 21, 18, 13 and sex chromosome aneuploidy in a pilot city in China. Hum Genomics.

[20] Teshima IE, Kalousek DK, Vekemans MJ, Markovic V, Cox DM, Dallaire L, Gagne R, Lin JC, Ray M, Sergovich FR (1992). Canadian multicenter randomized clinical trial of chorion villus sampling and amniocentesis. chromosome mosaicism in CVS and amniocentesis samples. Prenat Diagn.

[21] Gregg AR, Skotko BG, Benkendorf JL, Monaghan KG, Bajaj K, Best RG, Klugman S, Watson MS (2016). Noninvasive prenatal screening for fetal aneuploidy, 2016 update: a position statement of the American College of Medical Genetics and Genomics. Genet Medicine Official J Am College Med Genet.

[22] Teresa NS, Kao T, Mary EN (2017). Mosaic trisomy 16: what are the obstetric and long-term childhood outcomes?. Genet Med Official J Am College Med Genet.

